# Multivariate Statistical Approach for Nephrines in Women with Obesity

**DOI:** 10.3390/molecules26051393

**Published:** 2021-03-05

**Authors:** Ralitsa Robeva, Miroslava Nedyalkova, Georgi Kirilov, Atanaska Elenkova, Sabina Zacharieva, Błażej Kudłak, Natalia Jatkowska, Vasil Simeonov

**Affiliations:** 1 Department of Endocrinology, Faculty of Medicine, Medical University—Sofia, USHATE “Acad. Iv. Penchev”, 2, Zdrave Str., 1431 Sofia, Bulgaria; rali_robeva@yahoo.com (R.R.); drgkirilov@abv.bg (G.K.); atanaskae@gmail.com (A.E.); zacharieva67@gmail.com (S.Z.); 2 Department of Inorganic Chemistry, Faculty of Chemistry and Pharmacy, University of Sofia “St. Kl. Ohridski”, 1164 Sofia, Bulgaria; 3 Department of Analytical Chemistry, Faculty of Chemistry, Gdańsk University of Technology, 11/12 Narutowicza, 80-233 Gdańsk, Poland; blakudla@pg.edu.pl (B.K.); natjatko@pg.edu.pl (N.J.); 4 Department of Analytical Chemistry, Faculty of Chemistry and Pharmacy, University of Sofia “St. Kl. Ohridski”, 1164 Sofia, Bulgaria; vsimeonov@chem.uni-sofia.bg

**Keywords:** metanephrine, normetanephrine, diabetes, obesity, hypertension, cluster analyses, exploratory data analysis

## Abstract

Catecholamines are physiological regulators of carbohydrate and lipid metabolism during stress, but their chronic influence on metabolic changes in obese patients is still not clarified. The present study aimed to establish the associations between the catecholamine metabolites and metabolic syndrome (MS) components in obese women as well as to reveal the possible hidden subgroups of patients through hierarchical cluster analysis and principal component analysis. The 24-h urine excretion of metanephrine and normetanephrine was investigated in 150 obese women (54 non diabetic without MS, 70 non-diabetic with MS and 26 with type 2 diabetes). The interrelations between carbohydrate disturbances, metabolic syndrome components and stress response hormones were studied. Exploratory data analysis was used to determine different patterns of similarities among the patients. Normetanephrine concentrations were significantly increased in postmenopausal patients and in women with morbid obesity, type 2 diabetes, and hypertension but not with prediabetes. Both metanephrine and normetanephrine levels were positively associated with glucose concentrations one hour after glucose load irrespectively of the insulin levels. The exploratory data analysis showed different risk subgroups among the investigated obese women. The development of predictive tools that include not only traditional metabolic risk factors, but also markers of stress response systems might help for specific risk estimation in obesity patients.

## 1. Introduction

Stress is a state of threatened body homeostasis and effective physiological response mechanisms have been developed during evolution to counteract the intrinsic and extrinsic stressors [1]. Activation of hypothalamic-pituitary-adrenal axis and autonomic nervous system ensures a better survival chance in case of an acute threat, however, in the presence of chronic stress the same adaptive changes might become inadequate and they could contribute to the development of pathological conditions [1,2,3]. Catecholamines are the main stress hormones originating from different parts of chromaffine tissue. Epinephrine is produced by the adrenal medulla, while norepinephrine derives mainly from the peripheral sympathetic nervous system [4,5]. The 24-h urinary excretion of their metabolites metanephrine (MN) and normetanephrine (NMN) is used as a reliable test for evaluation of the adrenal gland activation, metabolism, and storage as well as sympathetic activity in men and women [5,6].

Catecholamines are well known physiological regulators of carbohydrate metabolism during stress, but their chronic influence might become pathological as seen in patients with catecholamine producing tumours. Our previous retrospective study revealed glucose tolerance disturbances in approximately 50% of individuals with pheochromocytomas or paragangliomas; almost 60% of them recovered normal glucose tolerance after successful tumor removal [7]. Nevertheless, the role of catecholamines is poorly investigated in patients with metabolic syndrome, prediabetes, and diabetes. Several studies have investigated the relationships between catecholamines and obesity but definitive conclusions are still lacking [4,6,8].

The present study is an example of the application of multivariate statistical methods based on method clusterization as well as the through principal component analyses for expediting the relation between the data obtained in the medical analysis. Therefore, the present study aims to investigate the role of the metabolites metanephrine and normetanephrine as markers of the adrenal and sympathetic stress response systems in overweight and obese women and to establish the possible associations between their 24-h urine concentrations and metabolic syndrome components.

## 2. Results

The main characteristics of diabetic (*n* = 26) and non-diabetic obese patients (*n* = 124) included in the study are presented on Table 1. Diabetic women were older than others and had higher levels of normetanephrine in comparison to non-diabetic patients. No differences in metanephrine levels were observed. The concentrations of metanephrine and normetanephrine were significantly related (r = +0.387, *p* < 0.001).

Among investigated non diabetic obese women 70 patients fulfilled the aforementioned MS criteria, while 54 individuals were with uncomplicated obesity (Table 2). The MS patients were older (age 41.30 ± 14.72 [41.00] vs. 34.04 ± 10.80 [33.00] years, *p* = 0.007) and more obese than the others (BMI 37.80 ± 5.74 [37.59] vs. 34.20 ± 5.78 [33.10] kg/m^2^, *p* < 0.001). The concentrations of nephrines did not differ between the patients with metabolic syndrome and those with uncomplicated obesity (78.93 ± 51.29 [63.00] vs. 91.74 ± 63.83 [70.50] µg/24 h, *p* = 0.301 for metanephrine; 328.27 ± 144.66 [299.50] vs. 295.02 ± 141.98 [304.00] µg/24 h, *p* = 0.252 for normetanephrine).

The concentrations of metanephrine and normetanephrine did not differ between the investigated obesity categories (*p* > 0.05 for all) (Supplementary Figure S1). However, extremely obese patients with BMI over 45 kg/m^2^ showed increased normetanephrine levels in comparison to less obese individuals (405.88 ± 132.46 [465.00] vs. 316.19 ± 150.11 [296.00] µg/24 h, *p* = 0.014).

Metanephrine levels were not associated with the age or waist circumference of the investigated obese patients (*p* > 0.05), but they were negatively related to the BMI (r = −0.166, *p* = 0.042). Normetanephrine concentrations were positively associated with the age (r = +0.248, *p* = 0.002) of the women, but not with their BMI or waist circumference (*p* > 0.05) (Supplementary Table S1).

The urinary normetanephrine levels were significantly increased in hypertensive patients compared to normotensive women (354.07 ± 150.06 [314.00] vs. 303.97 ± 148.05 [296.00] µg/24 h, *p* = 0.042), but not in patients with dyslipidemia or prediabetes (impaired glucose tolerance or impaired fasting glucose) (*p* > 0.05 for all). The presence of hypertension, dyslipidemia or prediabetes did not influence the 24 h-urine metanephrine concentrations (*p* > 0.05 for all).

Normetanephrine concentrations were associated with the levels of blood glucose at the first hour post glucose load (r = +0.196, *p* = 0.033), and with the total cholesterol (r = +0.220, *p* = 0.007), but not with the HOMA-IR, fasting glucose levels, HgbA1c, glucose levels at the second hour post load, insulin levels during glucose load or other lipid levels (*p* > 0.05). Similarly, metanephrine concentrations were positively related to the blood glucose levels at the first hour post glucose load (r = +0.215, *p* = 0.019), but not with the HOMA-IR, fasting glucose levels, HgbA1c, insulin levels during glucose load or other laboratory values (*p* > 0.05). Similar results were obtained after exclusion of patients on metformin and other antidiabetic drugs: metanephrine levels correlated with glucose levels at the first (r = +0.234, *p* = 0.031), and second hour post glucose load (r = +0.229, *p* = 0.028), while normetanephrine concentrations were associated only with the first hour glucose concentrations (r = +0.288, *p* = 0.007). The levels of 24 h-free nephrines were not associated with HOMA-IR or insulin levels (*p* > 0.05 for all). The same results were found also when patients on lipid-lowering therapy and antihypertensives were excluded/data not shown/.

**Figure 1:**
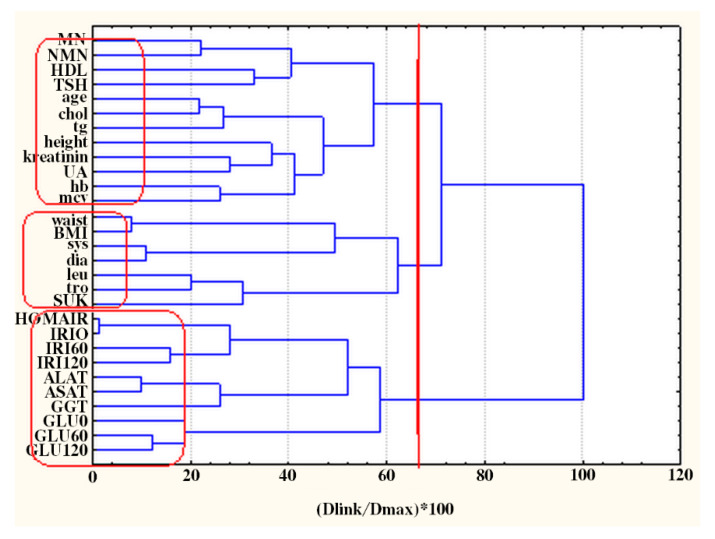
Hierarchical dendrogram for clustering of 29 variables. The included variables with their abbreviations are metanephrine (MN), normetanephrine (NMN), age, height, waist, body mass index (BMI), HOMA-IR, systolic blood pressure (sys), diastolic blood pressure (dia), leucocytes (leu), hemoglobin (hb), thrombocytes (tro), mean corpuscular volume (mcv), total cholesterol (chol), HDL-cholesterol (HDL), triglycerides (tg), fasting glucose (GLU0), glucose at the first hour post glucose load (GLU60), glucose at the second hour post glucose load (GLU120), creatinine (kreatinin), alanine aminotransferase (ALAT), aspartate aminotransferase (ASAT), gamma-glutamyl transferase (GGT), uric acid (UA), fasting insulin (IRI0), insulin at the first hour post glucose load (IRI60), insulin at the second hour post glucose load (IRI120), free urine cortisol (UFC), and thyroid stimulating hormone (TSH).

Increased NMN, but not MN levels were found in postmenopausal women compared to the premenopausal individuals (370.45 ± 139.61 [360.00] vs. 309.20 ± 154.75 [288.50] µg/24 h, *p* = 0.018). In the group of investigated women 30.6% were with irregular menstruation, 20.0% were hirsute and 25.0% of patients fulfilled the ESHRE PCOS criteria. MN and NMN were not different in patients with established PCOS, hirsutism or menstrual irregularities in comparison to patients with normal reproductive function (*p* > 0.05 for all). The variables linkage by hierarchical clustering in obese non-diabetic patients is shown in Figure 1.

**Figure 2:**
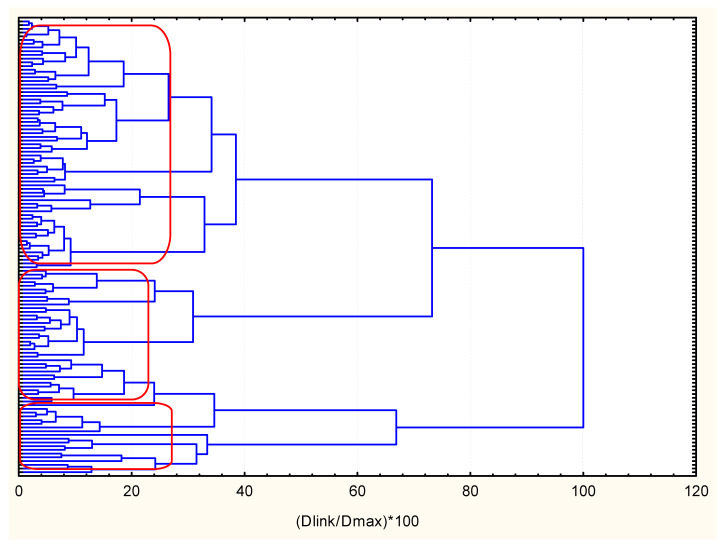
Hierarchical dendrogram for linkage of 124 patients.

Three major clusters were identified:

- C1 (GLU0, GLU60, GLU120, GGT, ASAT, ALAT, IRI0, IRI60, IRI120, HOMAIR)—glucose, insulin and liver function cluster;
- C2 (UFC, dia, sys, tro, leu, BMI, waist)—cortisol, blood pressure and obesity impact;
- C3 (MN, NMN, HDL, TSH, age, chol, tg, height, kreatinin, UA, hb, mcv)—nephrines, thyroid and blood quality cluster;

The same members of the three a priori sought clusters were obtained by the non- hierarchical K-means clustering. Objects (patients) linkage by hierarchical clustering is shown in Figure 2. Three clusters were identified and the non-hierarchical K-means clustering with hypothesis for formation a priori of three clusters was confirmed. C1 included 36 members, C2—40 members and C3—48 members.

**Figure 3:**
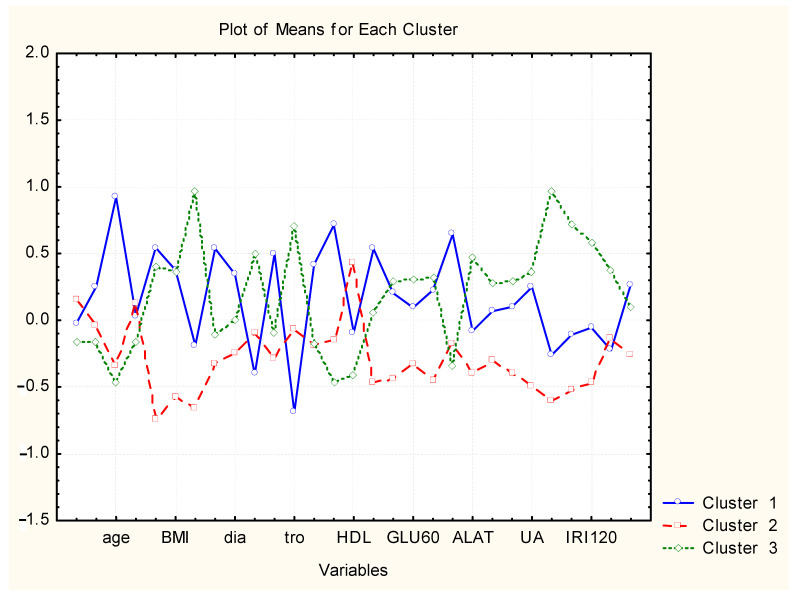
Plot of means of each descriptor (variable) for each identified cluster. The included variables (some of the variables are omitted for clarity) order in the axis as follows are presented with their abbreviations are metanephrine (MN), normetanephrine (NMN), age, height, waist, body mass index (BMI), HOMA-IR, systolic blood pressure (sys), diastolic blood pressure (dia), leucocytes (leu), hemoglobin (hb), thrombocytes (tro), mean corpuscular volume (mcv), total cholesterol (chol), HDL-cholesterol (HDL), triglycerides (tg), fasting glucose (GLU0), glucose at the first hour post glucose load (GLU60), glucose at the second hour post glucose load (GLU120), creatinine (kreatinin), alanine aminotransferase (ALAT), aspartate aminotransferase (ASAT), gamma-glutamyl transferase (GGT), uric acid (UA), fasting insulin (IRI0), insulin at the first hour post glucose load (IRI60), insulin at the second hour post glucose load (IRI120), free urine cortisol (UFC), and thyroid stimulating hormone (TSH).

Next Figure 3 represents the plot of means of each variable for each of the identified clusters.

The three different patterns of patients could be specified as follows:

*Cluster 1*: highest levels for *NMN*, *age*, *waist*, *BMI*, *sys*, *dia*, *hb*, *mcv*, *chol*, *tg*, *kreatinin*, *TSH*; lowest level for *tro*, *leu*. Probably, this might be described as a pattern of elderly patients with increased normetanephrine levels, severe central obesity, hypertension and lipid abnormalities.

*Cluster 2*: lowest levels of almost all parameters suggesting better health status.

*Cluster 3*: relatively high values for *waist and BMI*; highest levels for *HOMA-IR*, *tro*, *GLU0*, *GLU60*, *GLU120*, *ALAT*, *ASAT*, *GGT*, *UA*, *IRI0*, *IRI60*, *IRI120*, *UFC*. This might be described as a pattern of patients with pronounced insulin resistance, moderate central obesity, increased cortisol levels and hepatic disturbances.

**Figure 4:**
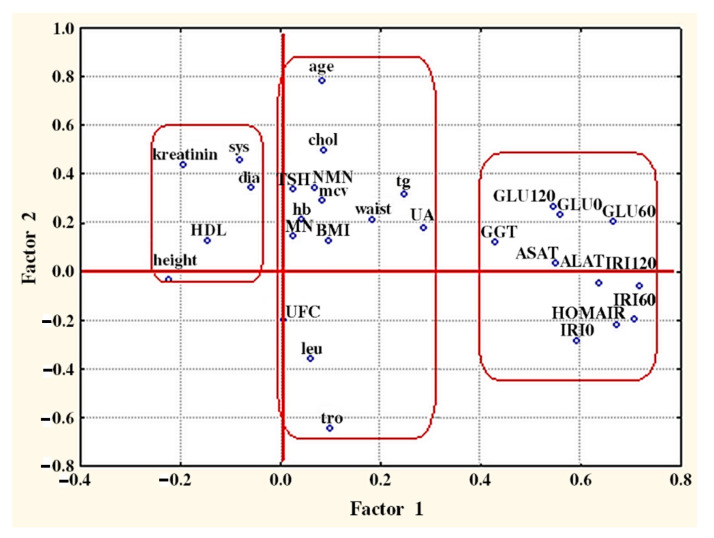
Plot of factor loadings (F1 vs F2). The included variables with their abbreviations are metanephrine (MN), normetanephrine (NMN), age, height, waist, body mass index (BMI), HOMA-IR, systolic blood pressure (sys), diastolic blood pressure (dia), leucocytes (leu), hemoglobin (hb), thrombocytes (tro), mean corpuscular volume (mcv), total cholesterol (chol), HDL-cholesterol (HDL), triglycerides (tg), fasting glucose (GLU0), glucose at the first hour post glucose load (GLU60), glucose at the second hour post glucose load (GLU120), creatinine (kreatinin), alanine aminotransferase (ALAT), aspartate aminotransferase (ASAT), gamma-glutamyl transferase (GGT), uric acid (UA), fasting insulin (IRI0), insulin at the first hour post glucose load (IRI60), insulin at the second hour post glucose load (IRI120), free urine cortisol (UFC), and thyroid stimulating hormone (TSH).

In general, the clustering of the variables is confirmed by factor analysis (Figure 4).

Factor analysis is well-known multivariate statistical approach for variables reduction and projection of multivariate data on a plane. The input variables are replaced by so called latent variables being linear combinations of the starting variables. Each one of the initial variables participates with a certain weight (loading) in the newly introduced latent factor (principal components) and this loading makes it possible to better interpret the physical meaning of the latent factors. Usually, each new principal component describes a part of the total variance of the system. The number of the new latent variables is determined either by the value of the total cumulative variance described (about 75%) or by the eigenvalue of each latent factor (significant factors are those with eigenvalue higher than 1). In our analysis three latent factors describe nearly 75% of the total variance and the biplot of the factor loadings of the first two latent factors is indicated in Figure 4. It is important to note that the grouping of the variables resembles that of cluster analysis.

## 3. Discussion

The study showed significant associations between the 24 h urine fractionated nephrines excretion and some metabolic disturbances in obese women. Several studies have shown interrelations between catecholamines and obesity [6,8,9,10]. A large study of Chinese men and women showed that gradual weight gain was accompanied by an increase of urinary norepinephrine secretion and a decrease of urine epinephrine secretion [6]. The authors hypothesized that the increased norepinephrine production in patients resulted from caloric excess and hyperinsulinemia leading to obesity and sympathetic nervous system activation. The subsequent increase of free fatty acids and glucose suppressed the adrenal medullar function to attenuate the metabolic disturbances [6]. Another large study revealed also reduced epinephrine adrenal secretion and storage in obese male and female volunteers in comparison to normal-weight individuals. However, urine normetanephrine levels did not differ between obese and lean participants [8]. Our study supported these results in a slightly different group of female patients with pronounced obesity. Metanephrine levels correlated negatively with the BMI, though no differences were observed between different obesity categories. Normetanephrine levels did not differ between overweight and obese women but they increased strongly in patients with morbid obesity. These results showed that opposite associations between the two components of sympathoadrenal system and obesity might depend on the obesity degree. The adrenal medullar dysfunction may facilitate the development of obesity, while the deposition of the excessive amount of fat tissue could stimulate the sympathetic nervous system activity.

Normetanephrine levels were significantly increased in patients with diabetes type 2, but not in patients with prediabetes (impaired fasting glucose or impaired glucose tolerance). These results supported the findings of Straznicky et al. who showed increased arterial norepinephrine levels in patients with uncomplicated diabetes in comparison to patients with impaired glucose tolerance [11]. However, age, concomitant medication and menopausal status might be potential confounders influencing the NMN levels in patients with diabetes type 2.

Both metanephrine and normetanephrine correlated positively with the blood glucose level at the first hour post glucose load but not with insulin levels during OGTT or HOMA-IR. Our results in obese women were in accordance with the data of Wang et al. who found increased glucose levels during oral glucose tolerance test in hypertensive normal-weight patients with plasma metanephrines in the upper ranges [12]. These associations are important for the clinical practice considering that the first-hour plasma glucose during OGTT is a potent predictor of type 2 diabetes, vascular complications, and mortality [13,14,15].

Catecholamines are insulin-counteracting hormones that could rapidly ensure glucose and other energy substrates during critical conditions [16]. However, the short- and long-term effects of epinephrine on carbohydrate metabolism might be contradictory. Epinephrine stimulates hepatic glycogenolysis and accelerates gluconeogenesis leading to a rapid increase in the plasma glucose concentrations [17]. On the opposite, it stimulates also insulin sensitivity and glucose uptake in the muscles thus slowly lowering glucose levels over time reviewed in [16]. Sympathetic nervous system influences the glucose metabolism through direct innervation of the liver, adipose and muscle tissues as well as indirectly through adrenal epinephrine and pancreatic glucagon stimulation [18]. An ingestion of carbohydrates is followed by a rapid increase of norepinephrine levels because of increased sympathetic nervous system activation in young and older patients. In the opposite, the epinephrine secretion pattern after carbohydrate load is characterized by an acute fall of plasma concentrations and their gradual rise subsequently [19,20]. Decreased adrenomedullar response to carbohydrates has been observed in elderly patients [19]. The postprandial decrease of epinephrine is blunted also in patients with obesity, thus the catecholamine disbalance might contribute to increased glucose levels after the carbohydrate load [10]. Accordingly, our data showed that both adrenomedullar dysfunction and increased sympathetic activity could modulate the postprandial glucose levels irrespective of insulin levels.

Despite the significant influence of the metanephrines on the BMI, postprandial blood glucose levels and hypertension their concentrations did not differ between non diabetic patients with metabolic syndrome and those with uncomplicated obesity. Therefore, exploratory data analysis was performed in order to reveal latent associations between the subjects and parameters of the investigation, and to describe the possible metabolic risk patterns of the obese non diabetic women. Using the described statistical approach, three phenotypes of obese non diabetic women were identified. The first cluster of similarities included elderly mostly postmenopausal patients with the highest levels of normetanephrine and unfavorable metabolic profile including increased blood pressure, dyslipidemia and visceral obesity. Probably, in that group of patients the age, postmenopausal status, metabolic disturbances and increased sympathetic activity determined the highest cardiovascular risk. The second phenotype included obese patients without significant metabolic risk factors or pronounced hyperinsulinemia and with low sympathetic activity ensuring the lowest cardiovascular and diabetic risk. The third cluster of patients included women with strong insulin resistance and or prediabetes. Despite the lack of other metabolic risk factors, the third phenotype was associated also with hepatic dysfunction and increased cortisol levels. The latter pattern described women with strongly increased risk of diabetes and non-alcoholic fatty liver disease, while the cardiovascular risk might be intermediate. The identified patterns emphasize on the heterogeneity of obesity and its complications.

Less than 20% of the included patients were with type 2 diabetes and therefore exploratory data analysis was not performed in that group. Other limitation of the study was the lack of plasma free metanephrine measurement in the investigated subjects. The observed correlations were weak, thus, metanephrine level might be considered as only one of the plenty of factors that could modulate the carbohydrate metabolism, and fat mass accumulation. Further study of diverse relationships between the stress response system and metabolic disturbances in pre- and postmenopausal obese women might be of great clinical importance.

In conclusion, our study explored the relations between the adrenal medullar dysfunction, sympathetic nervous system activity and obesity. Both metanephrine and normetanephrine levels were significantly associated with the early blood glucose increase after glucose load in obese women. The cluster analyses and PCA analysis revealed different risk subgroups among the investigated obese women. The development of predictive tools that include not only traditional metabolic risk factors, but also markers of the stress response systems might help for specific risk estimation in obesity patients.

## 4. Materials and Methods

### 4.1. Subjects and Study Protocol

A total of 150 overweight and obese women from Caucasian origin (age 18–72 years) were included in the present prospective study. They were recruited from the hospitalized patients and outpatients of our specialized Endocrinology Clinic seeking medical help for obesity, hypertension, dyslipidemia and/or carbohydrate disturbances. Patients with severe debilitating diseases, advanced liver or renal impairment, overt hypothyroidism, pheochromocytoma/paraganglioma, Cushing’s disease and/or cognitive dysfunction were excluded from the study. Men were not included in the study in order to avoid possible gender differences. Antidiabetic (mostly metformin), antilipemic and antihypertensive treatment of female patients was also recorded in detail.

Individuals underwent a complete general physical assessment, including height, weight, waist circumference and blood pressure measurement. Body mass index (BMI) was calculated according to the formula: BMI = weight/height^2^ (kg/m^2^). Patients with BMI ≥ 25 and/or a waist circumference ≥ 88 cm were considered overweight, while women with BMI ≥ 30 were classified as obese (grade 1 BMI 30.00–34.99; grade 2 BMI 35.00–39.99; grade 3 BMI 40.00–44.99; extremely obese BMI ≥ 45).

Metabolic syndrome was diagnosed in the presence of any three of the following five criteria: (1) Elevated waist circumference ≥ 80 cm; (2) Elevated triglycerides (TG) ≥ 1.7 mmol/L or drug treatment for elevated TG; (3) Decreased high-density lipoprotein cholesterol (HDL-ch) < 1.30 mmol/L or drug treatment for decreased HDL-ch; (4) Elevated fasting glucose ≥ 5.6 mmol/L or drug treatment for elevated glucose; (5) Systolic blood pressure ≥ 130 mmHg and/or diastolic blood pressure ≥ 85 mmHg and/or antihypertensive therapy [21]. The presence of prediabetes (impaired fasting glucose or impaired glucose tolerance) and diabetes was established according to current guidelines [22]. Reproductive history of participants including menstrual cycle regularity, signs of hyperandrogenism (acne, hirsutism), and menopause was thoroughly collected. A total of 28 % (*n* = 42) of patients were postmenopausal. The presence of polycystic ovarian syndrome according to the Rotterdam criteria in the premenopausal women was also recorded.

Blood and urine samples for laboratory assays including blood count, creatinine, liver enzymes, total cholesterol, HDL-ch, TG, hemoglobin A1c (in diabetic patients), thyroid stimulating hormone (TSH) and urine free cortisol (UFC) measurements were collected from the patients. In non-diabetic participants a two-hour, 75-g oral glucose tolerance test was also performed. Fasting glucose and immunoreactive insulin (IRI) levels were measured and both parameters were followed up at the first and second hours after the glucose load. Homeostatic model assessment index of insulin resistance (HOMA-IR) was calculated according to the equation: fasting serum insulin (µIU/mL) x fasting plasma glucose (mmol/L)/22.5.

Twenty-four hours’ urine samples for determining concentrations of metanephrine and normetanephrine were collected from all patients after specific instructions about necessary diet. The biochemical parameters were measured enzymatically by an automatic analyzer (Cobas Mira Plus; Hoffmann La Roche Basel, Switzerland)). IRI, UFC and TSH levels were determined through commercially available IRMA or RIA kits. Twenty-four-hour urinary metanephrines were measured by RIA with the following specific characteristics: for metanephrine (MN): reference range < 350 µg/24 h; analytical sensitivity 8 ng/mL, cross-reactivity < 0.02%; for normetanephrine (NMN) reference range < 600 µg/24 h; analytical sensitivity 22 ng/mL, cross-reactivity < 0.08% [7].

### 4.2. Statistical Analysis

Basic descriptive statistics, χ^2^ test and Fisher’s exact test were used for the analysis of dichotomous variables. Mean, standard deviation and median of the parameters were reported in the text. Spearman correlation analysis was performed to estimate possible associations. Differences between two groups were established with an independent sample t-test or Man-Whitney test according to normality of the distribution. Kruskal-Wallis test was used for multiple comparisons. The level of significance was set at the 0.05.

Additionally, exploratory data analysis was accomplished in order to reveal specific relationships between the aforementioned clinical parameters or between the obese patients. Separate multivariate statistical analysis was made in the groups of obese non diabetic women as described in a previous study [23]. The major aim was to identify clusters (patterns) of similarity between the variables in order to reveal possible hidden relationships between them and clusters (patterns) of similarity between the patients. Additionally, specific descriptors for each identified pattern of similarity between the patients were sought in order to determine the impact of each variable (test) in determining the health status of the patients. The multivariate statistical approaches used were mainly cluster analysis (hierarchical and non-hierarchical) and factor analysis.

The data set of the obese non diabetic women included a total of 124 patients and the parameters used for the statistical exploratory analysis were totally 29 [MN, NMN, age, height, waist, BMI, HOMA-IR, sys (systolic blood pressure), dia (diastolic blood pressure), leu (leucocytes), hb (hemoglobin), tro (thrombocytes), mcv (mean corpuscular volume), chol (total cholesterol), HDL (HDL-cholesterol), tg (triglycerides), GLU0 (fasting glucose), GLU60 (glucose at the first hour post glucose load), GLU120 (glucose at the second hour post glucose load), kreatinin (creatinine), ALAT (alanine aminotransferase), ASAT (aspartate aminotransferase), GGT (gamma-glutamyl transferase), UA (uric acid), IRI0 (fasting insulin), IRI60 (insulin at the first hour post glucose load), IRI120 (insulin at the second hour post glucose load), free urine cortisol (UFC), TSH]. The diabetic obese group included only 26 patients and therefore, the cluster analyses were not performed.

All calculations were by the use of the software packages MedCalc Statistical Software version 16.4.3 (MedCalc Software bv, Ostend, Belgium; https://www.medcalc.org; 2016) (accessed on 14 January 2021） and STATISTICA 8.0 [24].

## Figures and Tables

**Table 1:** Characteristics of overweight and obese female patients without diabetes mellitus type 2 (Non diabetic) compared to overweight and obese women with diabetes mellitus type 2 (DM2).

Characteristics	Non Diabetic		DM2		*p*
	Mean ± SD (Median)	N	Mean ± SD (Median)	N	
**Age (years)**	38.14 ± 13.60 (36.50)	124	44.96 ± 11.73 (41.50)	26	0.012
**Waist (cm)**	108.89 ± 14.10 (107.00)	124	117.35 ± 18.65 (118.00)	26	0.037
**BMI (kg/m^2^)**	36.23 ± 6.01 (35.17)	124	39.45 ± 11.44 (36.58)	26	0.174
**Systolic RR (mmHg)**	125.04 ± 12.60 (120.00)	124	138.08 ± 24.04 (130.00)	26	0.004
**Diastolic RR (mmHg)**	80.48 ± 7.84 (80.00)	124	84.23 ± 7.96 (80.00)	26	0.029
**Cholesterol (mmol/L)**	5.15 ± 1.06 (5.02)	124	5.59 ± 1.08 (5.52)	26	0.028
**HDL-cholesterol (mmol/L)**	1.34 ± 0.35 (1.29)	124	1.15 ± 0.32 (1.03)	26	0.006
**Triglycerides (mmol/L)**	1.44 ± 0.69 (1.31)	124	2.42 ± 2.53 (1.74)	26	0.001
**Creatinine (µmol/L)**	63.79 ± 9.59 (63.50)	114	62.27 ± 12.63 (62.50)	22	0.519
**ALAT (IU/L)**	23.09 ± 15.50 (16.75)	120	30.10 ± 14.40 (28.60)	25	0.004
**ASAT (IU/L)**	19.23 ± 9.43 (16.60)	118	22.60 ± 12.33 (19.76)	24	0.130
**GGT (IU/L)**	24.31 ± 17.77 (19.70)	116	45.94 ± 37.26 (32.29)	25	<0.001
**Uric acid (µmol/L)**	335.70 ± 70.34 (330.00)	106	359.00 ± 92.83 (344.00)	23	0.178
**Glucose 0′ (mmol/L)**	5.61 ± 0.71 (5.40)	124	8.69 ± 2.62 (8.05)	26	<0.001
**Glucose 60′ (mmol/L)**	7.58 ± 2.29 (7.15)	116			N.A.
**Glucose 120′ (mmol/L)**	5.82 ± 1.63 (5.50)	123			N.A.
**IRI 0′ (µIU/mL)**	17.69 ± 9.45 (15.50)	122	25.43 ± 14.45 (25.90)	16	0.024
**IRI 60′ (µIU/mL)**	106.53 ± 66.72 (92.45)	114			N.A.
**IRI 120′ (µIU/mL)**	55.80 ± 44.87 (45.70)	117			N.A.
**HOMA-IR**	4.46 ± 2.57 (3.60)	122	9.93 ± 7.53 (8.35)	21	<0.001
**HgbA1c (%)**			7.36 ± 1.81 (6.38)	24	N.A.
**TSH (µIU/mL)**	2.62 ± 1.46 (2.30)	119	2.78 ± 1.47 (2.40)	25	0.639
**Urine free cortisol (nmol/24 h)**	148.13 ± 112.51 (133.60)	121	136.20 ± 77.67 (139.50)	25	0.614
**MN (µg/24 h)**	84.51 ± 57.20 (69.00)	124	86.38 ± 53.62 (70.50)	26	0.815
**NMN (µg/24 h)**	313.79 ± 143.87 (304.00)	124	386.27 ± 169.42 (425.00)	26	0.032
**Hypertension**	38.7%	124	73.1%	26	0.001
**Antihypertensives**	29.8%	124	53.8%	26	0.019
**Metformin treatment**	29.0%	124	80.8%	26	<0.001

**Table 2:** Characteristics of overweight and obese non diabetic female patients without metabolic syndrome (MC) (Healthy obese) compared to overweight and obese non diabetic women with MS (Obese with MS).

Characteristics	Healthy Obese		Obese with MS		*p*
	Mean ± SD (Median)	N	Mean ± SD (Median)	N	
**Age (years)**	34.04 ± 10.80 [33.00]	54	41.30 ± 14.72 [41.00]	70	0.007
**Waist (cm)**	103.33 ± 13.87 [101.50]	54	113.17 ± 12.82 [113.00]	70	<0.001
**BMI (kg/m^2^)**	34.20 ± 5.78 [33.10]	54	37.80 ± 5.74 [37.59]	70	<0.001
**Systolic RR (mmHg)**	120.74 ± 10.57 [120.00]	54	128.36 ± 13.10 [130.00]	70	<0.001
**Diastolic RR (mmHg)**	78.24 ± 6.53 [80.00]	54	82.21 ± 8.36 [80.00]	70	0.007
**Cholesterol (mmol/L)**	5.08 ± 1.09 [4.89]	54	5.20 ± 1.04 [5.21]	70	0.241
**HDL-cholesterol (mmol/L)**	1.50 ± 0.31 [1.45]	54	1.21 ± 0.33 [1.13]	70	<0.001
**Triglycerides (mmol/L)**	1.10 ± 0.41 [1.07]	54	1.70 ± 0.75 [1.56]	70	<0.001
**Creatinine (µmol/L)**	63.71 ± 10.16 [63.5]	52	63.87 ± 9.18 [63.50]	62	0.931
**ALAT (IU/L)**	20.76 ± 13.86 [15.15]	52	24.87 ± 16.94 [18.25]	68	0.048
**ASAT (IU/L)**	18.21 ± 8.81 [15.95]	52	20.04 ± 9.89 [17.30]	66	0.265
**GGT (IU/L)**	20.85 ± 18.39 [15.65]	48	26.75 ± 17.04 [22.40]	68	<0.001
**Uric acid (µmol/L)**	307.16 ± 46.30 [315.00]	43	355.17 ± 77.32 [345.00]	63	<0.001
**Glucose 0′ (mmol/L)**	5.27 ± 0.49 [5.25]	54	5.88 ± 0.74 [5.90]	70	<0.001
**Glucose 60′ (mmol/L)**	6.97 ± 1.75 [6.80]	53	8.09 ± 2.57 [7.80]	63	0.027
**Glucose 120′ (mmol/L)**	5.18 ± 1.07 [5.10]	53	6.30 ± 1.81 [5.95]	70	<0.001
**IRI 0′ (µIU/mL)**	15.41 ± 8.55 [12.90]	53	19.45 ± 9.79 [17.70]	69	0.013
**IRI 60′ (µIU/mL)**	97.66 ± 66.96 [82.30]	53	114.23 ± 66.10 [102.30]	61	0.081
**IRI 120′ (µIU/mL)**	47.16 ± 44.57 [32.50]	53	62.96 ± 44.19 [53.00]	64	0.021
**HOMA-IR**	3.63 ± 2.10 [3.13]	53	5.26 ± 4.17 [4.93]	69	<0.001
**TSH (µIU/mL)**	2.50 ± 1.20 [2.35]	52	2.72 ± 1.64 [2.30]	67	0.757
**Urine free cortisol (nmol/24 h)**	132.15 ± 66.45 [115.00]	53	160.60 ± 137.42 [143.75]	68	0.234
**MN (µg/24 h)**	91.74 ± 63.83 [70.50]	54	78.93 ± 51.29 [63.00]	70	0.301
**NMN (µg/24 h)**	295.02 ± 141.98 [304.00]	54	328.27 ± 144.66 [299.50]	70	0.252
**Hypertension**	16.7%	54	55.7%	70	<0.001
**Antihypertensives**	9.3%	54	45.7%	70	<0.001
**Metformin treatment**	35.2%	54	24.3%	70	0.187

## Data Availability

Data available by request.

## Supplementary Materials

The following are available online, Supplementary Table S1: Correlation matrix including most important values. The included variables with their abbreviations are metanephrine (MN), normetanephrine (NMN), age, body mass index (BMI), systolic blood pressure (sys), diastolic blood pressure (dia), total cholesterol (chol), HDL-cholesterol (HDL), triglycerides (tg), fasting glucose (GLU0), glucose at the first hour post glucose load (GLU60), glucose at the second hour post glucose load (GLU120), fasting insulin (IRI0), insulin at the first hour post glucose load (IRI60), insulin at the second hour post glucose load (IRI120), free urine cortisol (UFC), and thyroid stimulating hormone (TSH). Supplementary Figure S1. Metanephrines and normetanephrines in patients with different degree of obesity.

## Sample Availability

Not available.
