# Supplementary material for: Multivariate Statistical Approach for Nephrines in Women with Obesity
Source: Molecules. 2021 Mar 5;26(5):1393. doi: 10.3390/molecules26051393 (PMC7961883; doi:10.3390/molecules26051393)
Supplement: Supplementary file 1 [file molecules-26-01393-s001.pdf]

**MN**	**r**<br>**p**<br>**n**										
**NMN**	**r**<br>**p**<br>**n**	0.387<br><0.0001<br>150									
**age**	**r**<br>**p**<br>**n**	-0.016<br>0.8485<br>150	**0.248**<br>**0.0022**<br>**150**								
**BMI**	**r**<br>**p**<br>**n**	**-0.166**<br>**0.0420**<br>**150**	0.122<br>0.1373<br>150	-0.031<br>0.7091<br>150							
**chol**	**r**<br>**p**<br>**n**	0.004<br>0.9610<br>150	**0.220**<br>**0.0068**<br>**150**	**0.446**<br>**<0.0001**<br>**150**	0.004<br>0.9587<br>150						
**HDL**	**r**<br>**p**<br>**n**	0.014<br>0.8670<br>150	0.082<br>0.3205<br>150	0.102<br>0.2147<br>150	**-0.262**<br>**0.0012**<br>**150**	0.110<br>0.1802<br>150					
**tg**	**r**<br>**p**<br>**n**	0.011<br>0.8974<br>150	0.084<br>0.3095<br>150	**0.298**<br>**0.0002**<br>**150**	**0.175**<br>**0.0322**<br>**150**	**0.439**<br>**<0.0001**<br>**150**	**-0.539**<br>**<0.0001**<br>**150**				
**sys**	**r**<br>**p**<br>**n**	-0.045<br>0.5874<br>150	**0.250**<br>**0.0020**<br>**150**	**0.282**<br>**0.0005**<br>**150**	**0.233**<br>**0.0042**<br>**150**	**0.238**<br>**0.0034**<br>**150**	-0.001<br>0.9910<br>150	**0.180**<br>**0.0272**<br>**150**			
**dia**	**r**<br>**p**<br>**n**	-0.072<br>0.3825<br>150	0.172<br>0.0357<br>150	0.075<br>0.3640<br>150	0.132<br>0.1077<br>150	0.131<br>0.1095<br>150	-0.041<br>0.6226<br>150	0.073<br>0.3728<br>150	**0.659**<br>**<0.0001**<br>**150**		
**GLU0**	**r**<br>**p**<br>**n**	-0.047<br>0.5691<br>150	0.104<br>0.2055<br>150	0.287<br>0.0004<br>150	0.123<br>0.1344<br>150	0.219<br>0.0071<br>150	-0.234<br>0.0039<br>150	0.281<br>0.0005<br>150	0.174<br>0.0329<br>150	0.072<br>0.3799<br>150	
**GLU60**	**r**<br>**p**<br>**n**	**0.215**<br>**0.0188**<br>**119**	**0.196**<br>**0.0328**<br>**119**	**0.191**<br>**0.0374**<br>**119**	0.096<br>0.3009<br>119	0.178<br>0.0532<br>119	-0.114<br>0.2165<br>119	**0.277**<br>**0.0023**<br>**119**	0.000<br>0.9996<br>119	-0.040<br>0.6660<br>119	**0.478**<br>**<0.0001**<br>**119**

**GLU120**	**r**<br>**p**<br>**n**	0.120<br>0.1788<br>127	-0.078<br>0.3857<br>127	0.112<br>0.2115<br>127	**0.175**<br>**0.0491**<br>**127**	0.008<br>0.9290<br>127	**-0.237**<br>**0.0073**<br>**127**	**0.314**<br>**0.0003**<br>**127**	0.123<br>0.1698<br>127	0.006<br>0.9500<br>127	**0.465**<br>**<0.0001**<br>**127**	**0.552**<br>**<0.0001**<br>**119**						
**IRI0**	**r**<br>**p**<br>**n**	-0.163<br>0.0523<br>143	-0.044<br>0.6019<br>143	-0.228<br>0.0062<br>143	0.396<br><0.0001<br>143	-0.042<br>0.6208<br>143	-0.389<br><0.0001<br>143	0.232<br>0.0054<br>143	0.023<br>0.7808<br>143	0.112<br>0.1828<br>143	0.214<br>0.0103<br>143	0.264<br>0.0038<br>118	0.194<br>0.0301<br>125					
**IRI60**	**r**<br>**p**<br>**n**	-0.063<br>0.4999<br>117	0.002<br>0.9843<br>117	0.003<br>0.9725<br>117	0.093<br>0.3181<br>117	0.082<br>0.3768<br>117	-0.170<br>0.0667<br>117	**0.213**<br>**0.0213**<br>**117**	-0.103<br>0.2684<br>117	-0.055<br>0.5532<br>117	**0.270**<br>**0.0033**<br>**117**	**0.417**<br>**<0.0001**<br>**117**	0.169<br>0.0679<br>117	**0.535**<br>**<0.0001**<br>**116**				
**IRI120**	**r**<br>**p**<br>**n**	0.053<br>0.5620<br>120	-0.094<br>0.3078<br>120	0.009<br>0.9188<br>120	0.015<br>0.8696<br>120	0.032<br>0.7252<br>120	**-0.287**<br>**0.0015**<br>**120**	**0.286**<br>**0.0015**<br>**120**	-0.062<br>0.5046<br>120	0.039<br>0.6745<br>120	**0.233**<br>**0.0104**<br>**120**	**0.520**<br>**<0.0001**<br>**117**	**0.642**<br>**<0.0001**<br>**120**	**0.516**<br>**<0.0001**<br>**119**	**0.581**<br>**<0.0001**<br>**117**			
**TSH**	**r**<br>**p**<br>**n**	0.006<br>0.9423<br>144	0.140<br>0.0939<br>144	-0.043<br>0.6091<br>144	**0.166**<br>**0.0470**<br>**144**	0.133<br>0.1121<br>144	0.099<br>0.2366<br>144	0.011<br>0.8975<br>144	0.158<br>0.0583<br>144	0.146<br>0.0808<br>144	0.061<br>0.4658<br>144	0.079<br>0.4062<br>114	0.079<br>0.3867<br>122	0.122<br>0.1539<br>137	-0.004<br>0.9701<br>112	0.026<br>0.7797<br>115		
**UFC**	**r**<br>**p**<br>**n**	0.097<br>0.2442<br>146	0.092<br>0.2703<br>146	-0.125<br>0.1343<br>146	0.023<br>0.7865<br>146	-0.058<br>0.4860<br>146	-0.102<br>0.2196<br>146	0.037<br>0.6547<br>146	0.119<br>0.1516<br>146	0.054<br>0.5137<br>146	0.009<br>0.9109<br>146	0.054<br>0.5629<br>117	0.062<br>0.4928<br>124	0.115<br>0.1793<br>139	0.150<br>0.1098<br>115	0.038<br>0.6850<br>117	-0.048<br>0.5694<br>141	
**Parameter**<br>**Spearman rank correlation coefficient**		**MN**	**NMN**	**age**	**BMI**	**chol**	**HDL**	**tg**	**sys**	**dia**	**GLU0**	**GLU60**	**GLU120**	**IRI0**	**IRI60**	**IRI120**	**TSH**	**UFC**

Supplementary table 1. Correlation matrix including most important values. The included variables with their abbreviations are metanephrine (MN), normetanephrine (NMN), age, body mass index (BMI), systolic blood pressure (sys), diastolic blood pressure (dia), total cholesterol (chol), HDL-cholesterol (HDL), triglycerides (tg), fasting glucose (GLU0), glucose at the first hour post glucose load (GLU60), glucose at the second hour post glucose load (GLU120), fasting insulin (IRI0), insulin at the first hour post glucose load (IRI60), insulin at the second hour post glucose load (IRI120), free urine cortisol (UFC), and thyroid stimulating hormone (TSH).

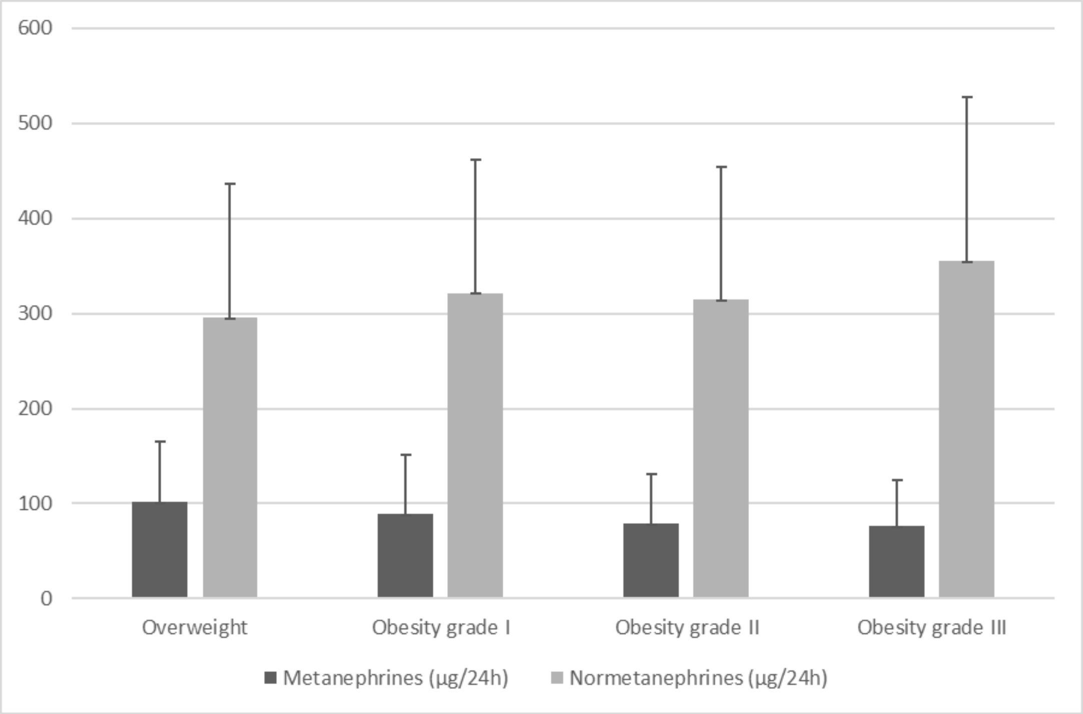

Supplementary figure 1. Metanephrines and normetanephrines in patients with different degree of obesity.
